# Vitamin D Supplementation Improves the Effects of the Rehabilitation Program on Balance and Pressure Distribution in Patients after Anterior Cervical Interbody Fusion-Randomized Control Trial

**DOI:** 10.3390/nu12123874

**Published:** 2020-12-18

**Authors:** Wojciech Skrobot, Ewelina Perzanowska, Katarzyna Krasowska, Damian J. Flis, Katarzyna P. Dzik, Wojciech Kloc, Jan Jacek Kaczor, Jędrzej Antosiewicz

**Affiliations:** 1 Department of Functional Diagnostics and Kinesiology, Gdansk University of Physical Education and Sport, 80-336 Gdansk, Poland; ewelinaliedtke@gmail.com (E.P.); katarzyna.krasowska@awf.gda.pl (K.K.); 2 Department of Physiology and Biochemistry, Gdansk University of Physical Education and Sport, 80-336 Gdansk, Poland; damian.flis@awf.gda.pl (D.J.F.); kasi.dzik@gmail.com (K.P.D.); jan.kaczor@awf.gda.pl (J.J.K.); 3 Department of Neurosurgery, Copernicus Hospital, 80-803 Gdansk, Poland; wk56rak@gmail.com; 4 Department of Neurology and Neurosurgery, University of Warmia and Mazury in Olsztyn, 10-719 Olsztyn, Poland; 5 Department of Bioenergetics and Physiology of Exercise, Medical University of Gdansk, 80-211 Gdansk, Poland

**Keywords:** vitamin D supplementation, ACIF, cervical stabilization, balance, Romberg test, postural stability, risk of falls, limits of stability

## Abstract

Study Design: A double-blinded, randomized controlled trial. Background: Surgery is effective in reducing pain intensity in patients with cervical disc disease. However, functional measurements demonstrated that the results have been not satisfactory enough. Thus, rehabilitation programs combined with the supplementation of vitamin D could play an essential role. Methods. The study recruited 30 patients, aged 20 to 70 years, selected for anterior cervical interbody fusion (ACIF). The patients were randomly divided into the placebo (Pl) and vitamin D (3200 IU D3/day) supplemented groups. The functional tests limits of stability (LOS), risk of falls (RFT), postural stability (PST), Romberg test, and foot pressure distribution were performed before supplementation (BS—week 0), five weeks after supplementation (AS—week 5), four weeks after surgery (BSVR—week 9), and 10 weeks after supervising rehabilitation (ASVR—week 19). Results. The concentration of 25(OH)D3 in the serum, after five weeks of supplementation, was significantly increased, while the Pl group maintained the same. The RFT was significantly reduced after five weeks of vitamin D supplementation. Moreover, a further significant decrease was observed following rehabilitation. In the Pl group, no changes in the RFT were observed. The overall postural stability index (OSI), LOS, and the outcomes of the Romberg test significantly improved in both groups; however, the effects on the OSI were more pronounced in the D3 group at the end of the rehabilitation program. Conclusions. Our data suggest that vitamin D supplementation positively affected the rehabilitation program in patients implemented four weeks after ACIF by reducing the risk of falls and improving postural stability.

## 1. Introduction

Patients with cervical disc disease often present with symptoms that include pain, sensory loss, motor loss, and reflex impairment. The consequence of these is a physical disability and a significant drop in quality of life [1]. Anterior cervical interbody fusion (ACIF) is a standard procedure for such problems; however, the outcome is strongly dependent on postsurgery rehabilitation [2]. Postoperative rehabilitation to increase the effects of cervical spine surgery by encouraging safe physical activities, improving function and reducing the fear of pain. Surgery has been shown to be very effective in pain reduction; however, in functions like balance, postural control, foot pressure distribution, and endurance, the progress is less evident [3].

Still, rules do not exist, and any guidance of time to conduct them initiate after an ACIF operation. Recently, it has been demonstrated that a self-directed home exercise program implemented immediately after surgery is effective in a short-term outcome [4]. Therefore, a rehabilitation program applied after surgery is crucial and usually focused on the improvement of cervical muscle endurance, strength, and postural control [3]. 

Vitamin D is an endogenous hormone synthesized in the skin from 7-dehydrocholesterol or taken from food. This vitamin is then metabolized in the liver by vitamin D-25-hydroxylase to 25-hydroxy vitamin D3 (25(OH)D3). 25(OH)D3 is converted by 1α-hydroxylase to 1,25-dihydroxy vitamin D3 (1,25(OH)2D3), which is an active form. Conversely, the serum concentration of 25(OH)D3 is considered to be a respectable marker of vitamin D status. If the concentration is lower than 30 ng/mL (75 nmol/L), it is considered inadequate by many scientists [5]. The kidneys and many other cell types possess the activity of 1α-hydroxylase and ability to synthesize 1,25(OH)2D3, including the skin, the human brain, prostate, white blood cells, and some others. The active form of vitamin D acts throughout its receptor (VDR), which is a transcriptional factor regulating around 900 genes [6]. Interestingly, VDR is present in the brain, skeletal muscles, and in most of the cells in the human body [7]. In agreement with this, skeletal muscle strength is associated with vitamin D status [8]. The anti-inflammatory properties of vitamin D have been also demonstrated [9]. All these properties make this vitamin an attractive remedy for patients after orthopedic surgery. A vitamin D deficiency is highly prevalent, and it is now considered a pandemic [10]. Its inadequacy is particularly common among orthopedic patients and osteoporosis. Besides, several studies have demonstrated the association between vitamin D deficiency, the spine, and joint morbidities [11,12]. 

Consequently, in the present study, we investigated the effects of vitamin D supplementation combined with 10 weeks of a supervised rehabilitation program in patients after ACIF surgery.

## 2. Materials and Methods 

### 2.1. Participants, Therapists, and Centers

**Figure 1:**
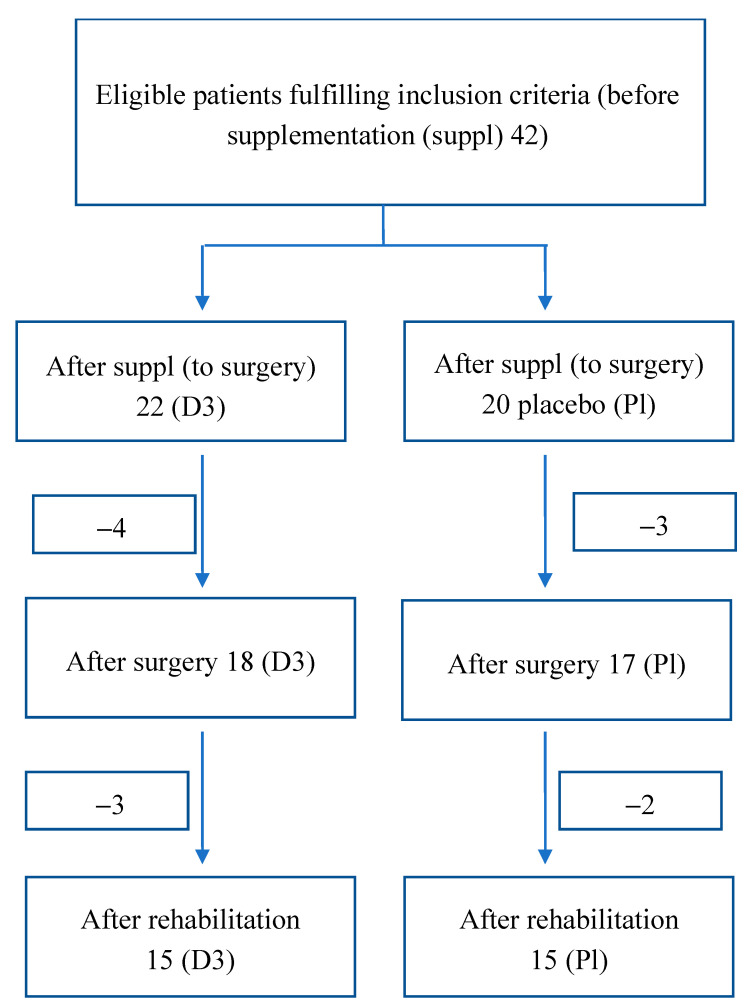
Participants flow diagram.

The study was a double-blinded, randomized controlled trial (Figure 1). 

At the beginning of the study, 42 patients were recruited and randomly selected to the vitamin D supplemented (D3) and placebo groups (Pl). After supplementation, 4 patients resigned in the D3 group and three in the P1 group.

In the further process after the operation, 3 people in group D3 resigned and, in group Pl, 2 people.

The reasons for resignation were very different; in three cases, it was due to the change of the type of spine surgery, as well as the presence of other diseases or colds at that time.

A total of 5 people withdrew after surgery before starting rehabilitation. It was mainly related to well-being and organizing rehabilitation closer to home (Figure 1).

Vitamin D supplementation 3200 IU daily for 5 weeks. The physiotherapists conducted the treatment of the patients in both groups. The independent physiotherapists performed functional tests. 

Patients were all coded, and the researchers, physiotherapists performing and reporting the statistical analyses, were unaware of the patients’ assignments.

Patients did not know what kind of supplement they received. All the patients gave full written informed consent to participate in the study and could leave the study at any time. 

Patients at COPERNICUS Hospital Neurosurgery Clinic in Gdansk, Poland were classified for ACIF surgery by a neurosurgeon clinical assessment and magnetic resonance imaging (MRI) scan.

The study group consisted of 30 people aged 31 to 68, 10 men and 20 women. Seventy-three percent of the respondents were people with secondary or vocational education, and 27% had higher education. Ten patients (5 women and 5 men) smoked cigarettes. The body mass index was 24.87 ± 5.83 and 25.67 ± 5.09 for the placebo and vitamin D groups, respectively. 

#### 2.1.1. Including Criteria

Ages between 20 and 70 years, neck pain, and/or upper limb neuropathy exceeding 12 months for which conservative treatment failed to improve a primary diagnosis of cervical spinal stenosis or degenerative disc disease, so the patient was selected for anterior cervical fusion with or without decompression.

#### 2.1.2. Excluding Criteria

Previous cervical fusion cervical ankylosing spondylitis rheumatoid arthritis and worsening of existing or appearance of new symptoms.

### 2.2. Functional Tests

Postural stability test (PST), limits of stability test (LOS), and risk of falls test (RFT) were used to assess control of balance. Before testing on a Biodex Balance System (BBS), a familiarization was performed, as described before [13]. Additional Romberg test and distribution force of pressure was conducted on a treadmill. Tests were performed four times: before supplementation (BS)—vitamin D3 or placebo, 5 weeks after supplementation 2 or 1 days before surgery (AS), 4 weeks after surgery (BSVR), and 10 weeks after supervised rehabilitation (ASVR).

### 2.3. Sample Collection 

Blood for biochemical research was collected four times: before supplementation (BS)—vitamin D3 or placebo, 5 weeks after supplementation 2 or 1 days before surgery ((AS), 4 weeks after surgery (BSVR), and 10 weeks after supervising rehabilitation (ASVR) in serum separation tubes (Becton Dickinson, Oxford, UK), The blood was collected at rest, while fasting, in the morning hours of 7:00–8:00 a.m. The samples for serum isolation were centrifuged at 2000× *g* and was stored at −80 °C until later analysis. Serum 25-OH Vitamin D3 was determined using the 25-OH Vitamin D total ELISA kit (DE1971, Demeditec Diagnostics, Kiel, Germany) according to the manufacturer’s instructions. The intra-assay coefficients of variability (CVs) and inter-assay CVs reported by the manufacturer were 2–8% and 4–9%, respectively. 

### 2.4. Postural Stability Test

The BBS (Biodex Medical System Inc., Shirley, NY, USA) allowed an objective evaluation of postural stability through three indexes: the Overall Stability Index (OSI), the Anterior-Posterior Stability Index (APSI), and the Medial-Lateral Stability Index (MLSI). During tests, the platform was stable. The participants were asked to step on the platform in a bipedal stance with bare feet and open eyes looking forward to the monitor (BSS monitor), while their hands are hanging by their sides (hand support was not permitted). They were asked to stand straight, not to change their feet position, and only sway their body when it was needed.

In these conditions, these indexes represent fluctuations around a zero point established before testing when the platform is stable [13]. Each measurement was taken on a static platform at intervals: 20 s of testing and 10-s breaks. Higher scores indicate poorer postural stability. 

### 2.5. Risk of Falls Test

The risk of falls test measures the same properties as the postural stability test; however, this is performed on an unstable platform. RFT consisted of three measurements at intervals: 20 s of testing and 10-s breaks, as described in the Operation and Service Manual of Biodex Medical Systems. Bilateral assessment of the dynamic stance was done for assessing the risk of falls; levels 6 to 2 were used. Before the testing, a familiarization procedure was performed. In this test, only the Overall Stability Index was evaluated. This index was calculated through the degree of oscillation of the platform, where the lower-score indices indicated a lower risk of falls [14].

### 2.6. Limits of Stability Test

The patients performed three trials of the LOS test, which involved shifting their body weight while standing on the stable platform to move a cursor on the screen from a central target to a peripheral blinking target [15].

During the test, the best results are obtained by patients who can obtained the maximum angle of a body from vertical without losing one’s balance. The Biodex Balance System provides scores for all eight directions, as well as an overall score. Higher scores indicated better performance. When the LOS is exceeded a fall, stumble, or step will ensue. 

### 2.7. Romberg Test

In the current study, treadmill Zebris (the Zebris FDM-T system (Zebris^®^ Medical GmbH, Isny im Allgäu, Germany) was used with sensors to measure the ground force of pressure. Patients were informed about the aim and methodological procedures of the test. The Romberg test served to assess balance and postural sway [16]. 

The subject kept a normal standing straight position, with his arms along his body and feet opened at an angle of 30 degrees to the front, heels 3 cm apart and narrow position [17]. 

The test was conducted with eyes closed (EC) during 30 s [18].

The outcomes of the Romberg test were gathered for the sway rate, center of pressure (CoP) path length, and ellipse sway area [19]. According to Kalron:

(1) the ellipse sway area (mm^2^), defined as a 95% confidence ellipse area for the mean movements of the center of pressure (CoP) anterior, posterior, medial, and lateral coordinates.
(2) the sway rate (mm/s), defined as the mean speed of movement of the CoP throughout the testing period.
(3) the CoP path length (mm), defined as the absolute length of the CoP path movements throughout the testing period.
(4) the average pressure distribution of the left and right feet expressed in body weight (%). Additionally, the bilateral pressure distribution asymmetry score was calculated as the absolute difference in pressure distribution between the legs. In a perfect symmetrical stance, this variable is zero [20].

The study was approved by the local institutional Bioethical Committee in Gdansk, Poland (No. NKBBN/120/2012) and conformed to the Declaration of Helsinki guidelines and was registered as clinical trial NCT03417700.

### 2.8. Data Analysis 

Statistical analysis was performed using Statistica 13.1 software (Statsoft, Tulsa, OK, USA). All values were expressed as the mean ± standard deviation (SD). The analysis of variance (ANOVA) in the repeated measures model, using the contrast method, was used for the analysis with *p* < 0.05. The study design was a two-arm randomized controlled trial with an allocation ratio of 1:1. An online software (https://www.graphpad.com/quickcalcs/randMenu/) was used to randomize the participants to the experimental group and, respectively, to the control group by the head of the project. 

### 2.9. Rehabilitation Protocol

After recrution as a part of the preparation to surgery, the patients were instructed by a physiotherapist how to move ergonomically before and after the surgery. Besides that, the patients were motivated to start activity without pain as soon as possible after the operation. 

The patients initiated a supervised rehabilitation protocol four weeks after ACIF surgery lasting 10 weeks (3 times a week). Physiotherapists were trained in rehabilitation protocols before the start of the exercises. 

During the early postoperative phase, strengthening exercises were performed while keeping the cervical spine in a neutral position to minimize strain on the fused/adjacent segment and, thereafter, to avoid breakage of the fusion device (Table 1).

## 3. Results

### 3.1. Vitamin D

**Figure 2:**
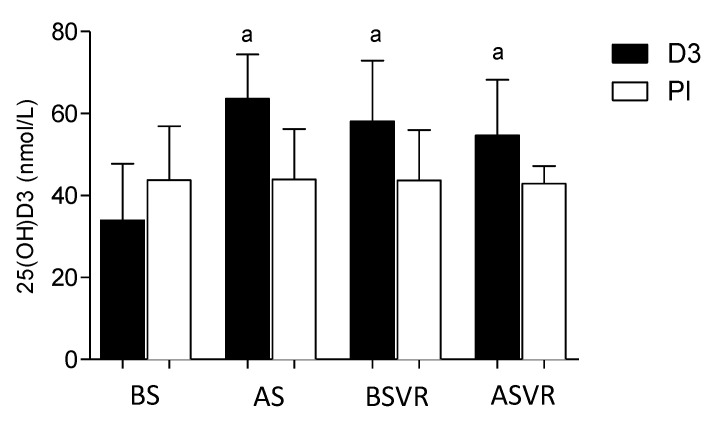
The concentration of 25(OH)D3 in the serum, after five weeks of supplementation, was significantly increased. Columns, mean; bars SD; and a, *p* < 0.05, significantly different compared with BS. BS—before supplementation, AS—5 weeks after supplementation, BSVR—4 weeks after surgery, and ASVR—10 weeks after supervised rehabilitation.

The concentration of the serum 25(OH)D3 did not differ between the groups at the beginning of the study. After five weeks of vitamin D supplementation (3200 IU/day), it significantly increased serum 25(OH)D3 in group D3, while, in the Pl group, the changes were not significant (Figure 2). During the 14 weeks of the study, the higher concentrations of 25(OH)D3 were maintained in the D3 group as compared to the starting level. 

### 3.2. Risk of Falls

**Figure 3:**
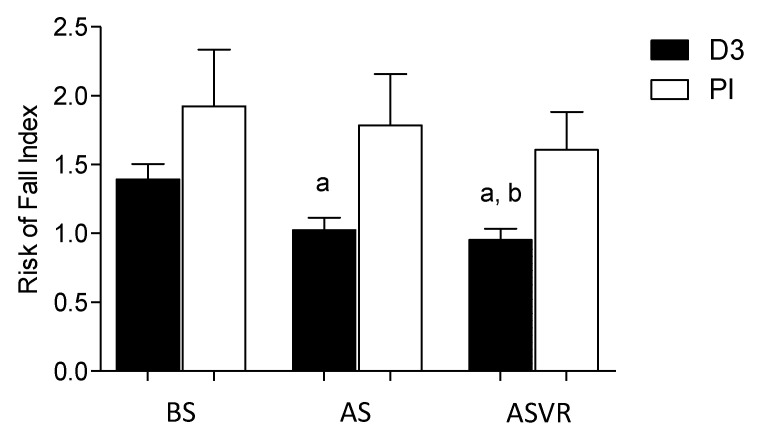
Vitamin D supplementation reduced the risk of falls of patients before anterior cervical interbody fusion (ACIF) surgery. The data are presented as the means and standard deviations (SDs). a, *p* < 0.05, significantly different compared with BS and b, *p* < 0.05, significantly different compared with Pl-ASVR.

In patients who were supplemented with vitamin D during the five weeks, significant improvement in the RFT was observed before ACIF operation. In the Pl group, no changes were found. Besides, after the surgery and 10 weeks of supervised rehabilitation, improvement in the RFT was observed only in the D3 group. The rehabilitation program caused some improvement in the Pl group, but it did not reach statistical significance (Figure 3). At the end of the study, after supervised rehabilitation protocols, significant differences between the groups were observed.

### 3.3. Postural Stability

**Figure 4:**
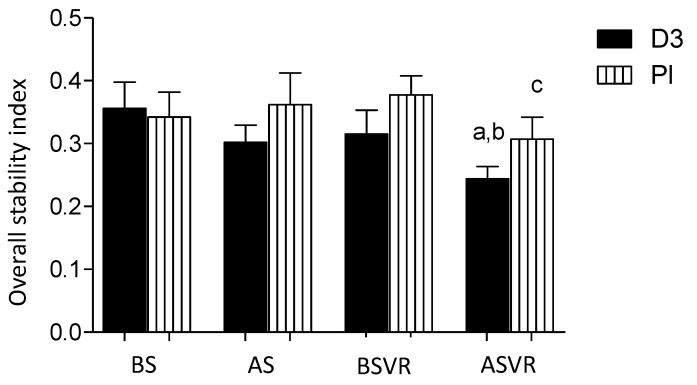
Vitamin D supplementation improves the effects of rehabilitation on the overall postural stability of patients after an anterior cervical interbody fusion surgery. Columns, mean and bars, standard deviations (SDs). a, *p* < 0.05, significantly different compared with the before supplementation (BS) control; b, *p* < 0.05, significantly different compared with the D3-BSVR group by one-way ANOVA; and c, *p* < 0.05, significantly different compared with the Pl-BSVR group.

The Postural Stability Test consists of indices: Anterior-Posterior Stability Index (APSI), Medial-Lateral Stability Index (MLSI) and Overall Stability Index (OSI), which is the composite of the MLSI and APSI. The lower scores of these indices indicate the improvement in postural control. Vitamin D supplementation before the ACIF operation had no effects on the OSI (Figure 4); however, the index significantly decreased in the D3 group after 10 weeks of the rehabilitation. Besides, in the placebo group, the significant improvement was observed only in the APSI after 10 weeks of rehabilitation (not shown). Moreover, the OSI were significantly better in the D3 group as compared to the Pl group at the end of the study. 

### 3.4. Limits of Stability

**Figure 5:**
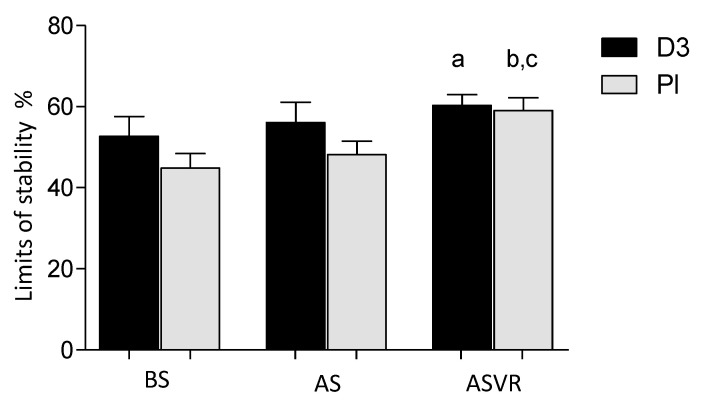
Vitamin D supplementation had no effects on the rehabilitation-induced improvement on the limits of stability of patients after anterior cervical interbody fusion surgery. Columns, mean and bars, standard deviations (SDs). a, *p* < 0.05, significantly different compared with the D3-BS; b, *p* < 0.05, significantly different compared with the Pl-BS; and c, *p* < 0.05, significantly different compared with and the Pl-after supplementation (AS) group, respectively by one-way ANOVA.

Five weeks of vitamin D supplementation had no effects on the limits of stability. Ten weeks of supervised rehabilitation significantly improved the LOS in both the D3 and Pl groups, and there were no differences between the groups (Figure 5).

A higher score of the Limits Stability Test compared to the initial level showed an improvement of posture balance control. The limits of stability, both in the vitamin D and placebo groups compared to the level before supplementation, after rehabilitation was significantly increased—a and b, *p* < 0.05. 

### 3.5. Ellipse Sway Area 

**Figure 6:**
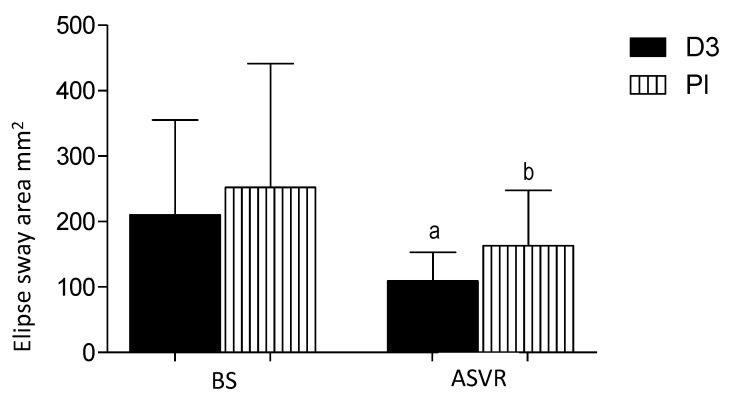
Vitamin D supplementation had no effects on the rehabilitation-induced improvement on the ellipse sway area (mm^2^) of patients after anterior cervical interbody fusion surgery. Columns, mean and bars, standard deviations (SDs). a, *p* < 0.05, significantly different compared with D3-BS and b, *p* < 0.05, significantly different compared with Pl-BS by one-way ANOVA.

The ellipse sway area (mm^2^) is defined as a 95% confidence ellipse area for the mean movements of the CoP anterior, posterior, medial, and lateral coordinates. After supervisor rehabilitation, the level of this marker significantly decreased in both the D3 and placebo groups compared to the baseline values (Figure 6). 

### 3.6. Sway Rate Center of Pressure (COP AV)

**Figure 7:**
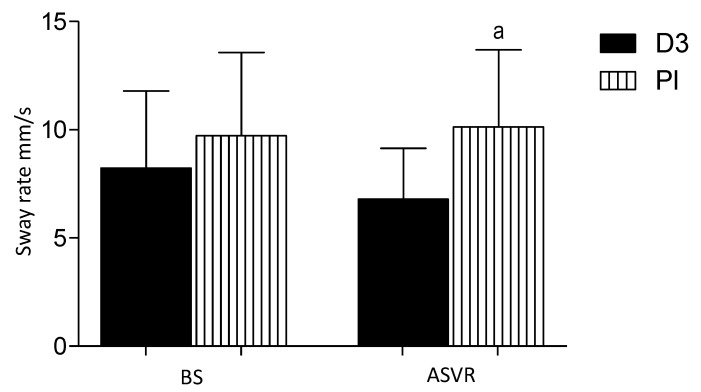
Vitamin D supplementation decrease sway rate (mm/s) in patients after anterior cervical interbody fusion surgery. Columns, mean and bars, standard deviations (SDs). a, *p* < 0.05, significantly different compared with ASVR D3 by one-way ANOVA.

The sway rate (mm/s) COP AV is defined as the mean speed of movement of the CoP throughout the testing period. After supervisor rehabilitation, there was a significant difference between the groups being lower in the D3 group. As compared to the beginning of the study, the deterioration of the results of the placebo group to the vitamin D group was observed; a, *p* < 0.005 (Figure 7).

### 3.7. CoP Path Length

**Figure 8:**
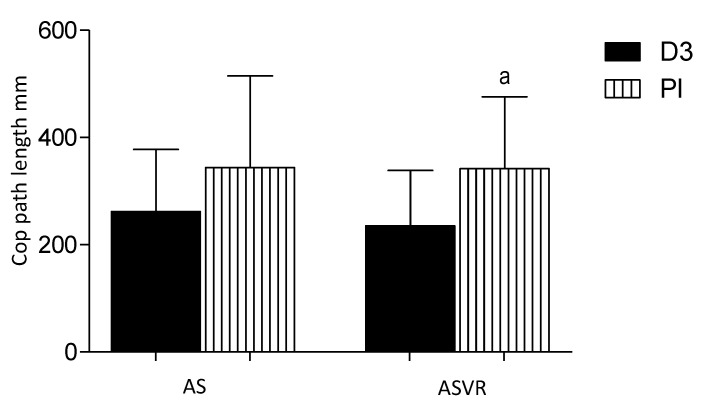
Vitamin D supplementation decreased the center of pressure (CoP) path length (mm) in patients after anterior cervical interbody fusion surgery. Columns, mean and bars, standard deviations (SDs). a, *p* < 0.05, significantly different compared with ASVR D3 by one-way ANOVA.

The CoP path length (mm) is defined as the absolute length of the CoP path movements throughout the testing period. After supervisor rehabilitation, there was a significant difference between the groups being lower in the D3 group (Figure 8).

The average pressure distribution of the left and right feet expressed in body weight (%) was also evaluated in the patients; however, the changes were not significant. 

## 4. Discussion

In the present study, we demonstrated that the vitamin D supplementation significantly improved the outcome of supervising rehabilitation in the D3 group who underwent ACIF surgery. Vitamin D supplementation was shown to exert positive effects on many aspects of human health, and many orthopedic patients were demonstrated to be vitamin D-deficient [21,22]. Despite this, we are not aware of any study where the effects of vitamin D on supervising the rehabilitation of cervical spine patients have been evaluated. Recently, some beneficial effects of vitamin D supplementation were observed during the functional recovery of patients after hip fractures and posterior interbody fusions [23,24]. Vitamin D has a pleiotropic function in the human body, including anti-inflammatory, and protects from skeletal muscle atrophy, improves proprioception, and many others [25]. It is worth noting that the five-week supplementation significantly increases 25(OH)D3 in the serum. 25(OH)D3 is considered to be a good marker of vitamin D status, as high concentrations (usually higher than 32 ng/mL) ensure the proper synthesis of 1,25(OH)2D3 in the target tissue. The presence of 25(OH)D3-1α-hydroxylase, the enzyme that converts 25(OH)D3 into 1,25(OH)2D3 in skeletal muscles, has been demonstrated [25]. However, up to today, it has not been well-defined what is the exact source of 1,25(OH)2D3 for skeletal muscles. The effects of 25(OH)D3 on skeletal muscle genes expression and muscle function were distinct when compared to 1,25(OH)2D3 [26]. Unfortunately, 1,25(OH)2D3 was not measured in this study; despite this, we found that a rise in 25(OH)D3 was associated with a lower risk of falls in patients even before the surgery. This data is in agreement with a previous study that demonstrated that vitamin D reduced the risk of falls in elderly subjects [26]. Impaired proprioception is one of the main factors that weakens postural control. Interestingly, a vitamin D deficiency was associated with poorer postural control and body sways [27]. Our data are partially in agreement with these observations. We demonstrated that 10 weeks of supervised rehabilitation decreased the risk of falls only in vitamin D-supplemented patients. Conversely, data from the LOS test showed improvement in both the Pl and D3 groups, which indicates that vitamin D supplementation did not modify the effects of rehabilitation. The LOS test measures the ability to shift the center of gravity toward the limits of stability in all directions, so its improvement indicates that the risk of falls decreases and quality of life increases. It is difficult to explain the difference in the results between the RFT and the LOS test. There is a tendency for better results in the LOS test in patients of the D3 group, which may indicate some positive effects. Additionally, the directional control, which is also evaluated in the LOS test, showed no differences between the group of unstable patients compared to healthy subjects [27]. On the contrary, our data indicated that the rehabilitation program improved the outcome of this test, which confirms its diagnostic value in patients after ACIF surgery. Moreover, the limits of stability are also known to decrease with age [28], which can also influence the results.

As mentioned above, the presence of VDRs in the human neurons, glial cells, and cortex have been demonstrated. Vitamin D deficiency in older people may contribute to failure in the central and peripheral nervous systems, including balance problems and the reduction of nerve conduction velocity [29]. Cervical spine receptors have important connections to the vestibular system, the organ of sight, and several connections with the areas of the central nervous system (CNS). A vitamin D deficiency, in addition to neck disorders, may contribute to the dysfunction of the upper spine proprioceptors and affect neuromuscular control and coordination. Besides, many studies have shown that the cervical spine has an impact on human balance [30]. Our data partially support this assumption, as supervised rehabilitation supported by vitamin D supplementation decreased the risk of falls and increased the overall stability. Additionally, the presence of VDR has been confirmed in the vestibular system [31], which plays an important role in stability control.

The importance of the muscles stabilizing the cervical spine, emphasizing the high content of spindles in them, especially in suboccipital muscles, as well as the function of the deep flexors of the cervical spine, additionally increase due to the type of the improvement program used, with vitamin D3 supplementation strengthening its effects. This is reflected in the reduction of deflections and increased stability in relation to CoP—posturography parameters, such as COP AV, ellipse sway area, and CoP path length.

One of the important aspects of vitamin D action is the ability to increase muscle cell regeneration and decrease their apoptosis [32], which may play an important role in the healing process after ACIF surgery. Besides, as mentioned before, vitamin D has anti-inflammatory action. The reduction of proinflammatory cytokines in circulation was shown after vitamin D supplementation [29]. Moreover, a study in rats demonstrated that vitamin D ameliorates skeletal muscle inflammation induced by exercise, decreasing the MAP kinase activation and tumor necrosis factor level [33].

These data became even more interesting in light of a previously published study that showed that neck-specific functions were not improved in patients in a six-year follow-up after surgery [34].

### Limitation of the Study

Certainly, the number of patients is a significant limitation of this study. Despite severe improvements in postural control in patients supplemented with vitamin D, we did not observe differences between the placebo and vitamin D groups at the end of the study in most of the studied parameters. Possibly with a higher number of patients, the differences could be revealed. Skeletal muscles have been shown to possess VDR and the ability to the synthesized the active form of vitamin D, but it is also possible that they may also depend on 1,25(OH)2D3 from the blood [35,36]. In support of this, skeletal muscle genes expression are correlated strongly with blood 1,25(OH)2D3 [26]. Moreover, it has been shown that circulating 1,25(OH)2D3 correlates better with skeletal muscle strength and muscle genes expression than 25(OH)D3 [26]. Remember that exercise-induced changes in vitamin D metabolism and formation of some metabolites with no classical vitamin D activity like antioxidant or anti-inflammatory can be expected [37]. Thus, knowing the blood concentration of 1,25(OH)2D3, 24,25(OH)2D3 and 3-epi-25(OH)D3, in addition to 25(OH)D3 in both groups, could help better to explain the effects of vitamin D supplementation on the outcome of the rehabilitation.

## 5. Conclusions

Our data suggest that preoperative supplementations with vitamin D positively affected the rehabilitation program in patients if implemented four weeks after ACIF surgery, reducing the risk of falls and improving postural stability. We assume that patients should be treated with vitamin D preoperatively, as well as after surgery, as a form of supporting the rehabilitation process, because of a better and faster recovery.

## Figures and Tables

**Table 1:** The details of the rehabilitation protocol.

Week	Intervention
I	Instruction about ergonomics behavior during daily activities; instruction about keeping right position with correction of a head, shoulder, and pelvic girdle; and, also, deep trunk stabilization was led. Maintaining a neutral posture movement of the upper and lower limbs was performed.
II–III	The same exercise as above, but more difficulty was conducted. Exercises with a head’s laser for control movement of the head was applied. Shoulder’s girdle stabilization, with additional control of the key parts of the body, was carried out. Proprioception exercises were led with opened and closed eyes. Exercises in the closed chain on the wall in a standing position were performed, which were prepared with plank and balance exercises.
IV	Balancing exercises with sensorimotor discs with the control of body swaying were conducted. Learning tensions of the deep cervical flexors was managed.
V	Increasing the number of isometric contractions of stabilizing muscles exercises and changes in the sequence and time performed of the movement were applied.
VI–VII	At the beginning of the sixth week, independent head movements in the range to the threshold of pain in the cervical spines of patients were performed. In the next weeks, an increased intensity and difficulty of the exercises were applied.
VIII–X	From the eighth week, patients started plank exercises. After ten weeks of supervised rehabilitation, patients were encouraged to continue their activities at home.

## Abbreviations

ACIF	Anterior cervical interbody fusion
OSI	Overall postural stability index
Pl	Placebo
LOS	Limits of stability
RFT	Risk of falls
PST	Postural stability
BS	Before supplementation
AS	After supplementation
BSVR	After surgery—Before supervising rehabilitation
ASVR	After supervising rehabilitation
25(OH)D_3_	25-hydroxy cholecalciferol
1,25(OH)_2_D_3_	1,25-dihydroxycholecalciferol
VDR	Vitamin D receptor
MRI	Magnetic resonance imaging
BBS	Biodex Balance System
CVs	Coefficients of variability
APSI	Anterior-Posterior Stability Index
MLSI	Medial-Lateral Stability Index
EC	Eyes closed
CoP	Center of pressure
SDs	Standard deviations
CNS	Central Nervous System
